# A Primate APOL1 Variant That Kills *Trypanosoma brucei gambiense*

**DOI:** 10.1371/journal.pntd.0004903

**Published:** 2016-08-05

**Authors:** Anneli Cooper, Paul Capewell, Caroline Clucas, Nicola Veitch, William Weir, Russell Thomson, Jayne Raper, Annette MacLeod

**Affiliations:** 1 Wellcome Trust Centre for Molecular Parasitology, College of Medical, Veterinary and Life Sciences, University of Glasgow, Glasgow, United Kingdom; 2 Department of Medical Parasitology, Langone School of Medicine, New York University, New York, New York, United States of America; Institute of Tropical Medicine, BELGIUM

## Abstract

Humans are protected against infection from most African trypanosomes by lipoprotein complexes present in serum that contain the trypanolytic pore-forming protein, Apolipoprotein L1 (APOL1). The human-infective trypanosomes, *Trypanosoma brucei rhodesiense* in East Africa and *T*. *b*. *gambiense* in West Africa have separately evolved mechanisms that allow them to resist APOL1-mediated lysis and cause human African trypanosomiasis, or sleeping sickness, in man. Recently, APOL1 variants were identified from a subset of Old World monkeys, that are able to lyse East African *T*. *b*. *rhodesiense*, by virtue of C-terminal polymorphisms in the APOL1 protein that hinder that parasite’s resistance mechanism. Such variants have been proposed as candidates for developing therapeutic alternatives to the unsatisfactory anti-trypanosomal drugs currently in use. Here we demonstrate the *in vitro* lytic ability of serum and purified recombinant protein of an APOL1 ortholog from the West African Guinea baboon (*Papio papio)*, which is able to lyse examples of all sub-species of *T*. *brucei* including *T*. *b*. *gambiense* group 1 parasites, the most common agent of human African trypanosomiasis. The identification of a variant of APOL1 with trypanolytic ability for both human-infective *T*. *brucei* sub-species could be a candidate for universal APOL1-based therapeutic strategies, targeted against all pathogenic African trypanosomes.

## Author Summary

African trypanosomes are protozoan parasites that affect both humans and animals in poor rural areas of sub-Saharan Africa, and are a major constraint on health and agricultural development. Disease control is principally dependent on the administration of drugs, which are old and largely unsatisfactory. Humans are naturally resistant to infection by most African trypanosomes species because of a lytic protein component in their blood, called APOL1. However, human-infective trypanosomes, *T*. *b*. *rhodesiense* in East Africa, and *T*. *b*. *gambiense* in West Africa, have evolved separate mechanisms to disarm this lytic protein and cause disease. Recently, variants of APOL1 were discovered in some primates that are able to kill the East African human disease-causing sub-species. These APOL1 variants form the basis of current attempts to create novel therapeutic interventions that can kill both animal and human-infective trypanosomes. In this study, we show that another variant of the same protein from a West African baboon species is able to kill, not only East African human-infective trypanosomes, but also the West African parasites, which causes the majority of human African trypanosomiasis cases. This new APOL1 variant could be a potential candidate for anti-trypanosomal therapies targeted at all pathogenic trypanosome species.

## Introduction

African trypanosomes continue to exert a significant barrier to agricultural production and rural development across sub-Saharan Africa [1]. Due to a primate-specific innate trypanolytic mechanism, the majority of trypanosome species are unable to infect man. However, two sub-species of *Trypanosoma brucei*, *T*. *b*. *rhodesiense* and *T*. *b*. *gambiense*, have evolved distinct processes to resist this lysis and cause the debilitating and often fatal human form of African trypanosomiasis, known as sleeping sickness. The West African *T*. *b*. *gambiense* parasite typically causes a chronic disease profile, while the zoonotic *T*. *b*. *rhodesiense* sub-species, located in Eastern and Southern Africa, results in a more rapidly progressing, acute infection [2,3]. Seventy-million people over an area of 1.55 million km^2^ are at risk of contracting either of the two human-infective sub-species [4].

Current anti-trypanosomal drugs for medical and veterinary administration are largely unsatisfactory due to high toxicity, difficult treatment regimens, and emerging resistance [5–7]. Decades of drug development for African trypanosomiasis has produced safer refinements of existing therapies [7,8] and a number of promising novel drug candidates [9–11], but as yet no new anti-trypanosomal therapy has successfully passed phase III clinical trials. Furthermore, the adaptive immune response of vertebrates is rendered largely ineffective by the trypanosome’s ability to cyclically evade detection through variant surface glycoprotein (VSG)-mediated antigenic variation [12,13], placing a significant hurdle in the path of vaccine development. Broad-spectrum, safe, easily administered, and effective therapies to treat African trypanosomiasis are therefore still needed. The recent discovery of primate serum proteins that are able to kill both animal and human-infective trypanosomes is now offering opportunities for novel therapeutic approaches [14,15].

It has been known for over a century that the serum of humans and a small number of other Catarrhine primates are highly toxic to most African trypanosome species [16,17]. The molecular basis of this innate immunity in man has been elucidated and centres on two trypanolytic serum complexes, Trypanosome Lytic Factor 1 (TLF-1) [18,19] and TLF-2 [20,21], which share the same core protein components: haptoglobin-related protein (HPR) and apolipoprotein L1 (APOL1). HPR bound to haemoglobin mediates TLF-1 endocytosis via the haem-scavenging, haptoglobin-haemoglobin receptor (HpHbR) on the trypanosome’s surface [22–25]. Difficulty in purifying TLF-2 *ex-vivo*, has hindered discovery of exactly how this complex is bound and internalised by the parasite but it is known that it does not require HpHbR [21,26]. Despite differences in uptake, both TLF-1 and TLF-2 utilize the same lytic component in the form of the ionic channel-forming protein, APOL1 [22,27,28]. Following internalization, APOL1 undergoes a pH-dependant conformational change in the endolysosome pathway which releases it from the TLF complex [29,30], and promotes insertion into parasite membranes [31,32]. The exact mechanism of APOL1-mediated lysis that follows remains to be elucidated. In one recent model APOL1 insertion was found to disrupt both lysosomal and mitochondrial membranes, inducing an apoptosis-like cell death [33]. In contrast, an alternative model proposes that endosome recycling of APOL1 to the neutral environment of the parasite’s plasma membrane accelerates cation-selective channel activity and promotes lysis by osmotic swelling [34].

The *Trypanosoma* parasites responsible for animal trypanosomiasis are rapidly killed by this innate defence system, whereas the human sleeping sickness parasites, *T*. *b*. *rhodesiense* and *T*. *b*. *gambiense*, are able to resist lysis. In *T*. *b*. *rhodesiense*, resistance is effected by the VSG-derived, serum resistance associated (SRA) protein [35,36] which binds to the C-terminal domain of APOL1 in the endolysosome pathway preventing channel-mediated lysis [27,37–39], plausibly by impeding correct membrane insertion of APOL1 [34,40].

The mechanism of human serum resistance in *T*. *b*. *gambiense* has taken longer to unravel. *T*. *b*. *gambiense* typically grows to very low parasitemia and is difficult to adapt to laboratory models. An additional complicating factor is that *T*. *b*. *gambiense* shows two distinct "groups" that differ in genotype and phenotype [41–44]. The classic, clonal *T*. *b*. *gambiense* type [45], labelled “group 1” and found in West and Central Africa, is the predominant human-infective sub-species, responsible for 97% of all reported human cases [46]. *T*. *b*. *gambiense* group 1 strains are invariably resistant even after prolonged passage in laboratory rodents [42,47] and the mechanism underlying this resistance appears multifactorial, with at least three independent contributing components so far identified. Firstly the reduction of TLF-1 uptake through reduced expression and polymorphism of the *HpHbR* receptor that reduces binding affinity [48–50]; secondly, expression of a VSG-related *T*. *b*. *gambiense*-specific glycoprotein (TgsGP) which is essential, but not sufficient, for resistance [51] and which may increase resistance to APOL1 pore-mediated lysis by stiffening trypanosomal membranes [52]; and thirdly, faster APOL1 degradation has been proposed, through the action of cysteine peptidase [52,53]. A second, more virulent type of *T*. *b*. *gambiense* was identified in Cote d’Ivoire and Burkina Faso in the 1980’s [42,44] and defined as “group 2”, but has since virtually disappeared and may now be extinct. Studies of the limited number of group 2 strains that have been isolated indicate that these parasites are closely related to West Africa *T*. *b*. *brucei* [41,43,44,54] and exhibit a variable human serum resistance phenotype, in a manner superficially similar to *T*. *b*. *rhodesiense* [42,47,48]. Although the underlying resistance mechanism remains elusive it does not appear to involve a reduction in TLF-1 uptake [48] or the *SRA* [55] or *TgsGP* gene [56,57].

Unlike humans and gorillas [58,59], from which they diverged around 25 million years ago [60], several members of the Cercopithecidae (Old World monkey) family appear intrinsically resistant to *T*. *b*. *rhodesiense* [58,59,61]. Both serum and APOL1 from the East African baboon species, *Papio hamadryas*, has been demonstrated to effectively lyse human-infective *T*. *b*. *rhodesiense* [14,58]. This difference in innate immunity between *Homo sapiens* and *P*. *hamadryas*, has been pinpointed to the position of a single amino acid in the baboon APOL1 C-terminus which prevents the parasite’s SRA protein from binding and neutralising APOL1 lytic activity [62]. Furthermore, a nearly identical mutation has now also been detected in the C-terminus of APOL1 variants of some humans with African ancestry whose serum exhibits lytic activity against *T*. *b*. *rhodesiense* but not *T*. *b*. *gambiense* [63].

This led to the hypothesis that as *T*. *b*. *gambiense* is found only in West Africa, another variant of APOL1 may exist in some West African primates that is able to kill *T*. *b*. *gambiense*. In this study we examined the serum and APOL1 protein of a West African baboon species, *Papio papio*, suggested to be refractory to *T*. *b*. *gambiense* infection, with the ability to eliminate parasites in a laboratory infection [64]. Here we demonstrate that serum and recombinant protein from the *P*. *papio* APOL1 ortholog lyses representative strains of all sub-species of *T*. *brucei* in an *in vitro* assay system. The identification of an APOL1 variant with broad trypanolytic ability against *T*. *brucei* sub-species, including the most prevalent *T*. *b*. *gambiense* type, may provide a potential reagent for the development of universal APOL1-based therapeutic agents.

## Methods

### *Trypanosoma brucei* stocks

Representative bloodstream form cell lines were selected for each subspecies from a collection at the University of Glasgow and have been previously described. STIB247 is a *T*. *b*. *brucei* strain originally isolated from a hartebeest in Serengeti, Tanzania in 1971 [65]. The *T*. *b*. *rhodesiense* strain EATRO98 was isolated by the East African Trypanosomiasis Research Organization (EATRO) from a human in Nyanza, Kenya in 1961 [66]. *T*. *b*. *gambiense* group 2 strain STIB386 (MHOM/CI/78/TH114) was originally isolated in 1978 from an infected patient in Côte d'Ivoire [67]. ELIANE (MHOM/CI/52/ITMAP 2188) is a *T*. *b*. *gambiense* group 1 strain isolated from a human in Côte d’Ivoire in 1952 [68]. Additional *T*. *b*. *gambiense* group 1 strains tested were human isolates, PA (MHOM/CG/80/ITMAP1843/PA) from Republic of the Congo in 1975 [43], BIM (MHOM/CM/75/ITMAP1789/BIM) from Cameroon in 1975 [43], and TOBO (MHOM/CI/83/DAL596/TOBO) and S1/1/6 RI from Côte d'Ivoire in 1983 [69] and 2002 [70], respectively. All bloodstream form culture lines were maintained *in vitro* in modified HMI9 medium [71] supplemented by 1.5 mM glucose, 1 mM methyl cellulose, 250 μM adenosine, 150 μM guanosine and 20% foetal bovine serum (FBS). Expression of the *SRA* human serum resistance gene in *T*. *b*. *rhodesiense* EATRO98 was maintained under selection with 1% normal human serum. Ectopic expression of functional *T*. *b*. *brucei HpHbR* in ELIANE was previously generated using the tubulin-targeting *TbbHpHbR* pTub-phelo construct (strain ELIANE *TbbHpHbR*^*-/+*^) [51], and maintained under phleomycin selection. Bloodstream form isolates BIM and S1/1/6 RI were grown from stabilate in donor BALB/C mice (Harlan, United Kingdom) and trypanosomes purified from blood by differential centrifugation as previously described [72]. Cells were maintained as for bloodstream culture cells lines at 37°C in 5% CO_2_ for up to 24 hours until use. All animal procedures were carried out in accordance with the Animals (Scientific Procedures) Act of 1986. Subspecies classification for *T*. *b*. *gambiense* group 1 strains was confirmed by a positive PCR result for the *T*. *b*. *gambiense* specific glycoprotein (*TgsGP*) gene and *T*. *b*. *rhodesiense* by a positive PCR result for the subspecies-specific serum resistance-associated (*SRA*) gene, as previously described [48]. *T*. *b*. *brucei* and *T*. *b*. *gambiense* group 2 strains were confirmed by a combination of negative *SRA*/*TgsGP* PCR results, the human serum sensitivity phenotype and their microsatellite genetic profile [73].

### Serum stocks

Sera Laboratories International, UK, provided pooled adult *P*. *papio* baboon serum derived from two individuals. Additional *P*. *papio* baboon serum, derived from a single adult male individual, was provided by Matrix Biologicals, UK. Normal human serum was obtained from a consented human donor and subject to appropriate ethical approval. The APOL1 protein levels in all serum samples are unquantified.

### Serum resistance assays

Trypanosomes were diluted to 5 x 10^5^ parasites per ml in modified HMI9, with the addition of human serum or *P*. *papio* serum serially diluted in foetal bovine serum (FBS), or FBS only, to a total concentration of 20%. Assays were performed in a final volume of 200 μl in a standard 96 well plate at 37°C in a CO_2_-equilibrated incubator. The number of viable motile trypanosomes was quantified at 24 hours by haemocytometer counts under the microscope in triplicate, for at least three independent experiments. The percentage viability of parasites in human or *P*. *papio* serum was normalised relative to the FBS control for each cell line to account for inherent differences in strain growth rate. Dose–response curves and IC_50_ values were determined using GraphPad Prism software (version 7.0).

### Cloning and expression of recombinant APOL1

The *H*. *sapiens* (accession no. CCDS13926.1) or *P*. *papio* (accession no. KC197810) APOL1 open reading frame (ORF) was synthesised and supplied by GeneArt life technologies in an Invitrogen Gateway-compatible entry vector. The entry vector containing the *APOL1* cDNA sequence, minus the N-terminal signal peptide (*H*. *sapiens*, residues 28–398; *P*. *papio*, residues 28–288) was cloned into pDest17 destination vector, which added an N-terminal 6xHis-tag, and transformed into BL21- AI competent *E*. *coli*. Protein expression was induced using 0.2% L-Arabinose for 16 hours at 37°C. Cells were lysed with urea lysis buffer (8 M urea, 20 mM Tris-HCl, 0.5 M NaCl, 5 mM imidazole, pH 8) and the cellular detritus removed by centrifugation at 5000g for 15 minutes. A small aliquot was removed for analysis by SDS-PAGE and Western blot with 1:5000 HRP-conjugated mouse anti-His antibody (Qiagen) and the remainder was used for protein purification under denaturing conditions. Denatured 6x His-tagged APOL1 protein was purified by passing the cell lysate through a gravity-flow Ni-Sepharose column (Gravitrap, GE Healthcare), and washing several times with urea lysis buffer pH 8 supplemented with increasing concentrations of Imidazole (5 mM-50 mM). Finally, bound protein was eluted with urea lysis buffer pH 8 containing 500mM imidazole. The eluate was dialyzed overnight against 20mM acetic acid and 0.05% tween and concentrated using 10,000 MW Vivaspin columns (Sartorius). Purity and concentration of the final purified protein was checked using a Qubit fluorometer (Thermo fisher) and SDS-PAGE (S1 Fig), then the concentration adjusted to 1 mg/ml and stored in aliquots at 4°C.

### Recombinant APOL1 lysis assay

To assess survival in recombinant APOL1, trypanosomes were diluted to 5 x 10^5^ parasites per ml in modified HMI9 containing 20% FBS and incubated with a dilution series of recombinant human or *P*. *papio* APOL1. The recombinant APOL1 was formulated in protein-free buffer (20mM acetic acid, 0.05% tween) and added in a volume of 10 μl to a final assay volume of 200 μl in a standard 96 well plate. A control containing an equivalent volume of protein-free buffer was also prepared. Assays were performed at 37°C in a CO_2_-equilibrated incubator, and the number of viable motile trypanosomes in each well was quantified at 24 hours by haemocytometer counts under the microscope in triplicate for at least three independent experiments. Cell counts in recombinant APOL1 were compared to control wells containing protein-free buffer only to determine percentage survival. In each assay, cells were incubated in 20% normal human serum as a positive control. Dose–response curves, IC_50_ values and one-way ANOVA were determined using GraphPad Prism software (version 7.0). Where indicated, trypanosomes were pre-incubated with 10 mM ammonium chloride (NH_4_Cl), a weak base, for 30 minutes at 37°C to reverse acidification of the endolysosome system prior to the addition of recombinant APOL1.

### APOL1 localisation immunofluorescence assays

Samples for IFA were prepared as follows. All incubation steps unless stated otherwise were performed in a humidor at room temperature. Bloodstream form trypanosomes were diluted in HMI9 medium containing 20% FBS at a concentration of 10^6^ parasites/ml and incubated with 50 μg/ml purified recombinant *H*. *sapiens* or *P*. *papio* APOL1 for two hours at 37°C. After this period, cells were washed once in serum-free HMI9 medium, and settled onto glass slides before fixing in 1% paraformaldehyde for 10 minutes. Samples were permeabilised using 0.1% Triton X-100 in PBS for 20 minutes then incubated in blocking solution (2% BSA in PBS) for 20 minutes. After washing three times in PBS, slides were incubated for 40 minutes with 1:500 mouse anti-p67 antibody (gift from Jay Bangs, Department of Microbiology and Immunology, University at Buffalo, NY, USA) in blocking solution. Washes were repeated and then primary antibody was detected using 1:1000 goat anti-mouse AlexaFluor594 secondary antibody (Life technologies) incubated for 40 minutes in blocking solution. To detect His-tagged APOL1 slides were washed three times in PBS and then incubated for 40 minutes with 1:500 AlexaFluor488 mouse anti-penta-His antibody (Molecular Probes, Invitrogen) in blocking solution. Following a final three washes the cells were treated with 50% glycerol, 0.1% DAPI, 2.5% 1, 4-diazabicyclo [2.2.2] octane (DABCO) in PBS, protected with a coverslip and sealed with acetone. Slides were imaged using the Deltavision Core system and SoftWorx package (Applied Precision) with standard filter sets (DAPI/FITC/Texas-Red and Light transmission). Approximately 30 serial sections through each trypanosome were taken for each filter. The images were composited and the brightness, contrast and color levels normalised between samples and exposures using the ImageJ software package (US National Institute of Health).

### Ethics statement

The University of Glasgow ethical review board approved the use of human serum in this study. The human serum volunteer gave written informed consent.

## Results

### *P*. *papio* serum is lethal to *T*. *b*. *rhodesiense* and *T*. *b*. *gambiense* groups 1 and 2

**Fig 1:**
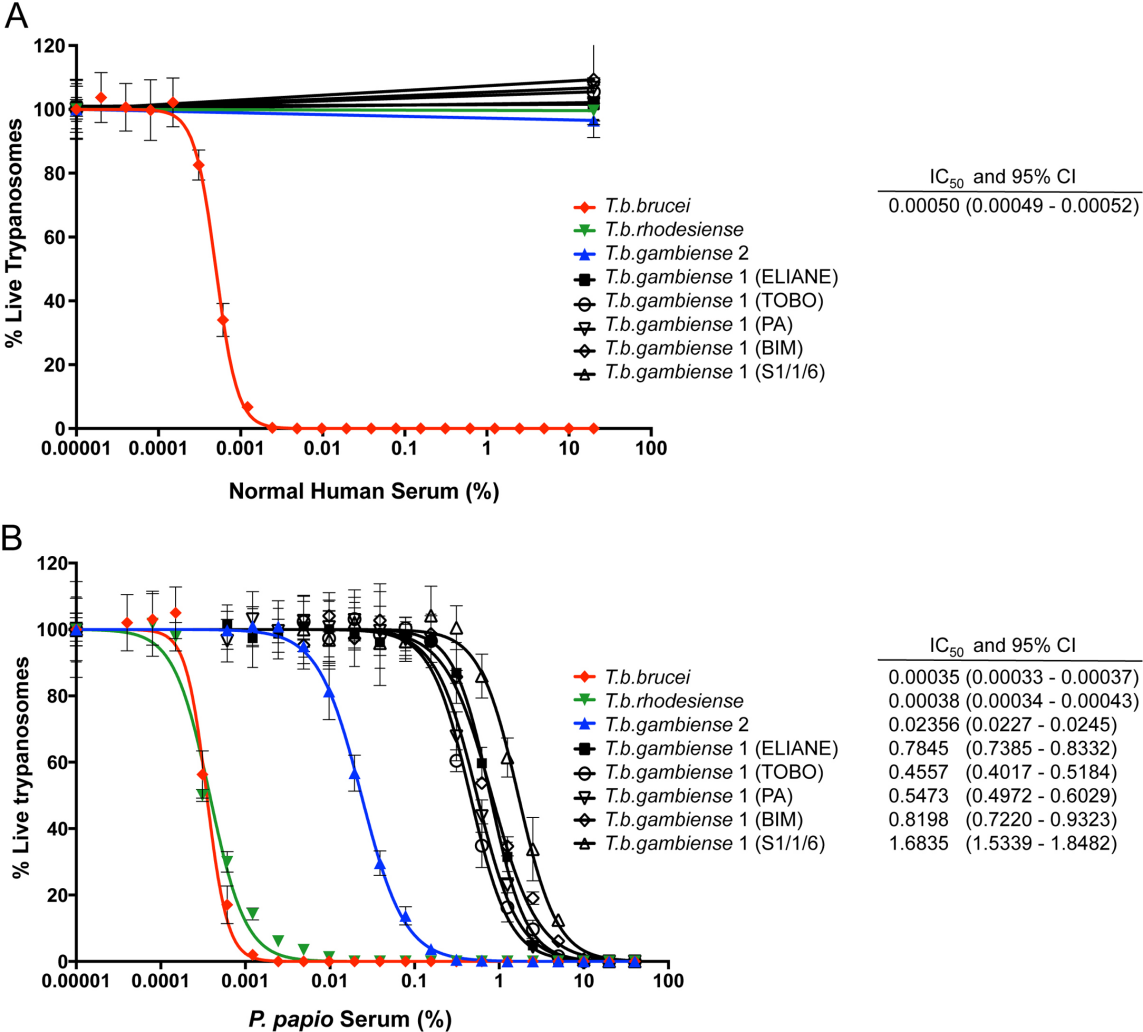
Titration of the trypanolytic activity of Human (*H*. *sapiens*) and Guinea baboon (*P*. *papio)* sera against representative examples of the *T*. *brucei* sub-species. The percentage of viable trypanosomes was determined following a 24-hour exposure to serial dilutions of (A) Human (*H*. *sapiens*) or (B) Guinea baboon (*P*. *papio)* sera. Representative *T*. *brucei* sub-species strains were tested: *T*. *b*. *brucei* (strain STIB247), *T*. *b*. *rhodesiense* (strain EATRO98), *T*. *b*. *gambiense* group 2 (strain STIB386), and *T*. *b*. *gambiense* group 1 (strains ELIANE, TOBO and S1/1/6 [Côte d'Ivoire], PA [Republic of the Congo], and BIM [Cameroon]). Mean percentage cell survival ± SD is expressed relative to FBS control, calculated from three independent experiments. Dose–response curves and IC_50_ values with 95% confidence intervals (CI) were determined using GraphPad Prism software version 7.

Trypanolytic activity against the human-infective East African *T*. *b*. *rhodesiense* sub-species has been demonstrated for sera from several members of the Cercopithecidae family, including baboons, mandrills and sooty mangabeys [14,37,58,59]. To date however, no primate has been identified with lytic activity against West African *T*. *b*. *gambiense* parasites. To determine the trypanolytic ability of serum from the West African Guinea baboon, *P*. *papio*, representative examples of the different *T*. *brucei* sub-species, were incubated for 24 hours *in vitro*, with a dilution series of *P*. *papio* or human serum. The strains selected included five different isolates of classic *T*. *b*. *gambiense* group 1, the cause of 97% of reported HAT cases [46], from a number of different disease foci in West Africa. As illustrated in Fig 1A, normal human serum efficiently lysed *T*. *b*. *brucei* bloodstream parasites (IC_50_; 0.0005%) in a 24 hour assay, but not strains of the human-infective *T*. *b*. *rhodesiense* or *T*. *b*. *gambiense* subspecies. In contrast, *P*. *papio* (pooled sera) was completely lytic to all tested strains, including both *T*. *b*. *gambiense* group 1 and 2 isolates, at concentrations ≥ 10% (Fig 1B). The sensitivity of *T*. *b*. *brucei* to *P*. *papio* pooled serum (IC_50_; 0.00035%) was comparable to that of *T*. *b*. *rhodesiense* (IC_50_; 0.00038%). *T*. *b*. *gambiense* group 1 and 2 strains however, were killed significantly less potently, with an IC_50_ approximately 70-fold (IC_50_; 0.024% serum, *T*. *b*. *gambiense* group 2) or 2000-fold (IC_50_; 0.46–1.68% serum, *T*. *b*. *gambiense* group 1) higher than that of the other sub-species, although still at a sub-physiological concentration. The trypanolytic activity of *P*. *papio* was also confirmed against a smaller collection of *T*. *brucei* strains using an alternative source of *P*. *papio* sera derived from a single male individual, which killed *T*. *b*. *gambiense* at a lower concentration > 2% (S2 Fig), presumably reflecting variation between individual animal samples.

### Lytic activity of *P*. *papio* recombinant APOL1

**Fig 2:**
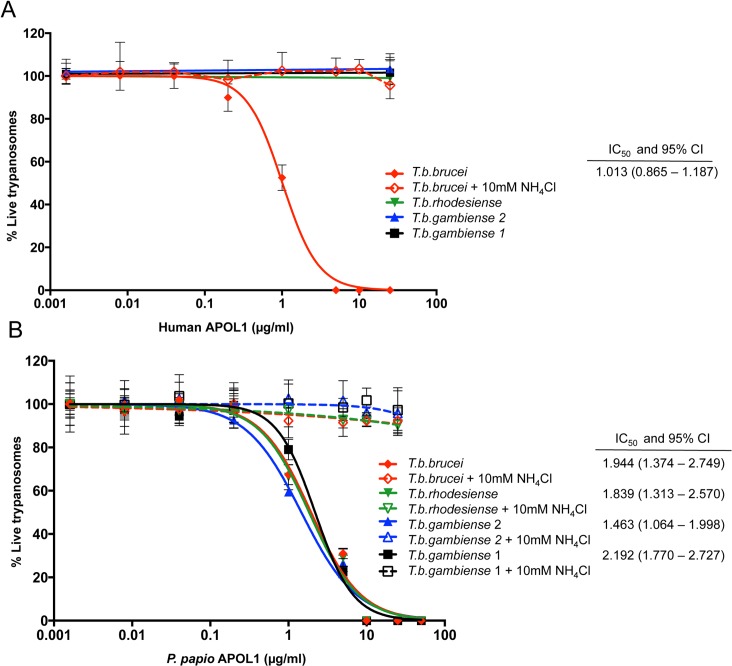
Trypanolytic activity of recombinant APOL1. The percentage of viable trypanosomes was determined following a 24-hour exposure to media containing serial dilutions of A) recombinant Human APOL1 protein and B) recombinant *P*. *papio* APOL1 protein. Representative *T*. *brucei* sub-species strains were tested: *T*. *b*. *brucei* (strain STIB247), *T*. *b*. *rhodesiense* (strain EATRO98), *T*. *b*. *gambiense* group 1 (strain ELIANE), and *T*. *b*. *gambiense* group 2 (strain STIB386). Additional assays were performed with different strains of *T*. *b*. *gambiense* group 1 and are provided in S4 Fig. The mean percentage cell survival ± SD, relative to protein-free control, was calculated from at least three independent experiments. Dose–response curves and IC_50_ values with 95% confidence intervals (CI) were determined using GraphPad Prism software version 7. APOL1-mediated lysis of each isolate was prevented by the inclusion of an acidotropic agent (10 mM NH_4_Cl) in the assay.

APOL1 has been demonstrated to be the lytic factor in normal human serum [22,27,28], and *T*. *b*. *rhodesiense-*lytic orthologs of APOL1 have now been identified in the serum of a number of Old World monkey species, including species of the *Papio* baboon genus [14,37,58]. Furthermore this lytic activity of Papio APOL1 against *T*. *b*. *rhodesiense* has been demonstrated to be the result of a single polymorphism [62]. We therefore hypothesize that the broad lytic ability of *P*. *papio* may be attributable to a functional variant of this protein. Sequenced *APOL1* cDNA was used as a template for the production of recombinant variants of *P*. *papio* and human APOL1 protein (S3 Fig-Amino acid alignment). Representative strains of the different *T*. *brucei* sub-species were incubated in the presence of purified *P*. *papio* and human recombinant protein to determine if APOL1 alone had demonstrable trypanolytic ability. Titrated human recombinant APOL1 protein completely lysed *T*. *b*. *brucei* parasites after 24 hours (IC_50_; 1.013 μg/ml), at concentrations comparable to the physiological levels of APOL1 reported for normal human serum [74–76], but had no lytic effect on strains of the human serum resistant parasites, *T*. *b*. *rhodesiense*, *T*. *b*. *gambiense* group 1 or *T*. *b*. *gambiense* group 2 (Fig 2A). In contrast, recombinant *P*. *papio* APOL1 protein exhibited trypanolytic activity against representative strains of all *T*. *brucei* sub-species (Fig 2B with additional *T*. *b*. *gambiense* group 1 strains assays provided in S4 Fig). Furthermore, strains of all sub-species tested appeared equally susceptible to the effect of recombinant *P*. *papio* APOL1, with no significant difference in IC_50_ observed (one-way ANOVA, F (3, 24) = 1.741, *p* = 0.19). Notably, as has been observed for human APOL1, this lytic activity is inhibited by the addition of the acidotropic agent ammonium chloride to the assay (Fig 2A and 2B). Ammonium chloride is a weak base that raises endolysosomal pH, thereby preventing pH-dependant conformational changes to APOL1 that are predicted to be essential to efficient ion-channel mediated lysis [32,34,77]. This corresponding inhibition of APOL1-mediated lysis for both orthologs is further indicative of a conserved mechanism of action. In summary these assays demonstrate that the *P*. *papio* APOL1 ortholog in isolation exhibits trypanolytic ability against all tested examples of the human-infective *T*. *brucei* sub-species. Although there may be other, as yet uncharacterized factors that contribute to the lytic ability of *P*. *papio* serum, the APOL1 ortholog is a significant trypanolytic component.

### *T*. *b*. *gambiense* group 1 HpHbR reduces sensitivity to *P*. *papio* serum lysis

A reduced sensitivity to lysis was observed for both the predominant *T*. *b*. *gambiense* group 1 and minor group 2 strains, relative to *T*. *b*. *brucei* and *T*. *b*. *rhodesiense*, when incubated with *P*. *papio* **serum**, but not recombinant APOL1 **protein**. We postulated that for *T*. *b*. *gambiense* group 1, this difference might be the result of disparity in the rate of uptake of APOL1 versus APOL1–containing trypanolytic factors by these parasites. In normal human serum, HPR bound to haemoglobin, acts as ligand to facilitate TLF-1 uptake via the *T*. *brucei* HpHbR receptor [23,78]. However, a defining feature of *T*. *b*. *gambiense* group 1 strains is a decrease in TLF-1 internalisation as a result of reduced *HpHbR* expression and a conserved L210S substitution that reduces the binding affinity of HpHbR for its ligand [50,79]. Reduced TLF uptake via HpHbR contributes to the invariant human serum resistant phenotype of these parasites, although alone is insufficient to impart resistance to human serum [78] due to the existence of other speculated receptors for TLF-1 [80,81], and the additional TLF-2 particle in human serum for which the uptake mechanisms remain unknown [21,82,83]. In contrast, recombinant APOL1 is internalised by non-specific fluid phase endocytosis and trafficked through the endolysosome pathway, thus completely circumventing the HpHbR receptor [27,48].

**Fig 3:**
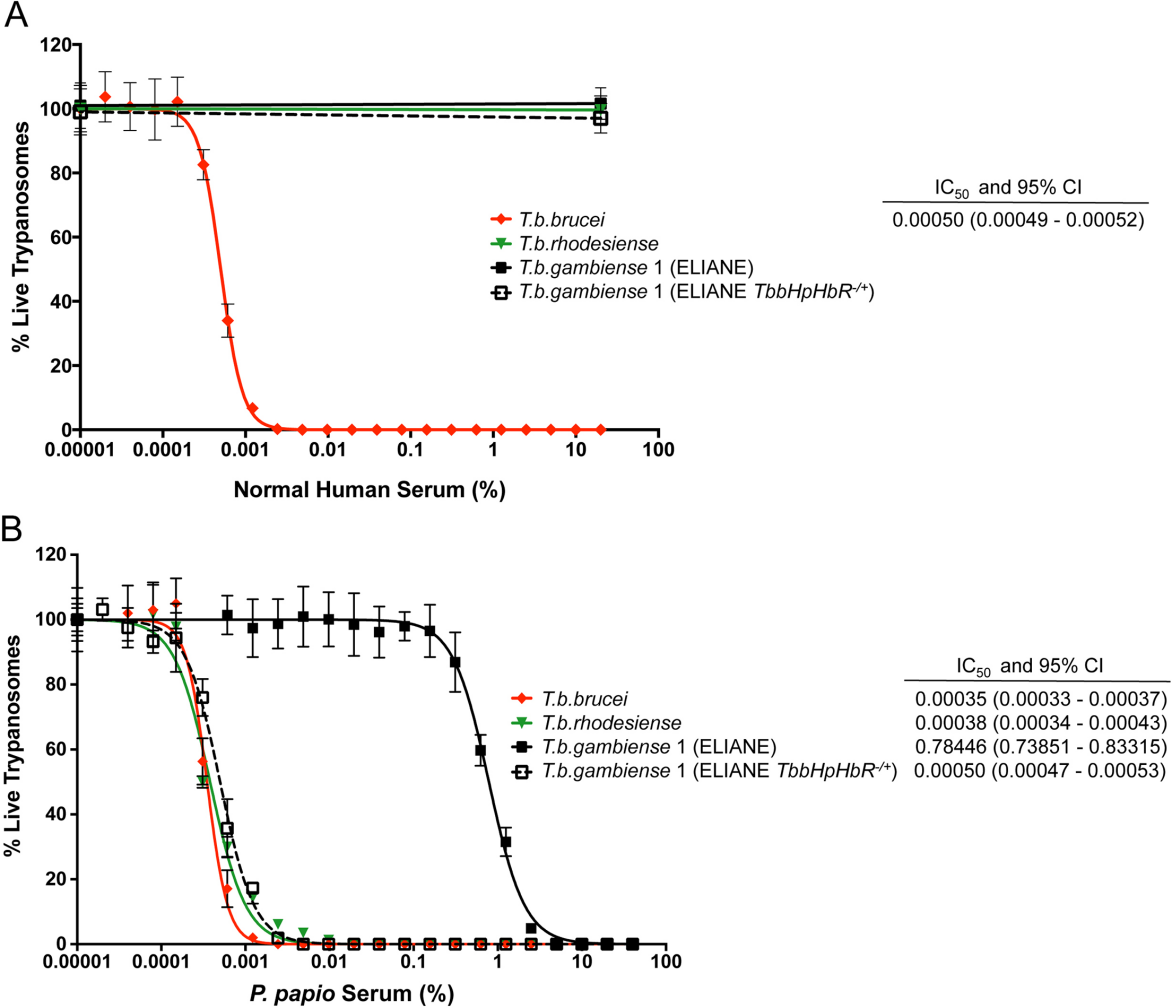
Expression of *T*. *b*. *brucei HpHbR* in *T*. *b*. *gambiense* group 1 increases sensitivity to *P*. *papio* serum lysis. The percentage of viable trypanosomes following a 24-hour exposure to serial dilutions of A) human serum and B) *P*. *papio* serum was determined for *T*. *b*. *gambiense* (strain ELIANE) and *T*. *b*. *gambiense* expressing a functional *T*. *b*. *brucei HpHbR* receptor (strain ELIANE *TbbHpHbR*^*-/+*^) alongside representative *T*. *brucei* sub-species strains *T*. *b*. *brucei* (STIB247) and *T*. *b*. *rhodesiense* (EATRO98). Mean percentage cell survival ± SD is expressed, relative to FBS control. Dose–response curves and IC_50_ values with 95% confidence intervals (CI) were determined using GraphPad Prism software version 7.

The number of molecules in the TLF complex and its exact structural composition in baboon serum is currently unresolved, but a representative baboon species, *P*. *hamadryas*, has been demonstrated to have similar constitutive components (HPR and APOL1) to human TLF [14]. As *T*. *b*. *gambiense* group 1 parasites have a reduced uptake of human TLF-1 but the other subspecies do not we postulated that a similar mechanism could reduce the uptake of *P*. *papio* TLF particles by *T*. *b*. *gambiense* group 1 strains, which is corrected by direct incubation in recombinant APOL1 protein. To investigate this we repeated the serum resistance assays using a *T*. *b*. *gambiense* ELIANE strain expressing a functional *T*. *b*. *brucei* HpHbR receptor (ELIANE *TbbHpHbR* ^*-/+*^), that was previously generated by our laboratory and demonstrated to take up comparable amounts of TLF-1 to *T*. *b*. *brucei* [51]. As previously observed, expression of the functional *T*. *b*. *brucei* HpHbR receptor alone was insufficient to convert the phenotype of *T*. *b*. *gambiens*e to human serum sensitivity and this clone (*TbbHpHbR* ^*-/+*^ *T*. *b*. *gambiense*) retains full resistance to normal human serum (Fig 3A). However, it exhibits a 1000-fold increased sensitivity to *P*. *papio* serum (relative to the wild-type *T*. *b*. *gambiense* group 1 ELIANE strain), producing an IC_50_ value (0.0005%) comparable to that observed for the *T*. *b*. *brucei* and *T*. *b*. *rhodesiense* sub-species (Fig 3B and S2 Fig). Taken together, the serum and APOL1 assays indicate that diminished TLF uptake via the HpHbR receptor, rather than higher innate resistance to *P*. *papio* APOL1-mediated lysis underlies the increased resistance to *P*. *papio* serum observed for *T*. *b*. *gambiense* group 1 strains.

In *T*. *b*. *gambiense* group 2, in contrast, an as yet uncharacterised HpHbR–independent mechanism/s determines human infectivity. *T*. *b*. *gambiense* group 2 strains, including the STIB386 isolate used in this study, have been shown to express the *HpHbR* gene at level comparable with *T*. *b*. *brucei*, with no demonstrable reduction in TLF-1 uptake [48]. Consequently, the reduced sensitivity to *P*. *papio* serum lysis, but not APOL1 protein, also observed for these HpHbR-functional parasites, further indicates that important differences exist in the cell biology of between *T*. *b*. *gambiense* group 2 and *T*. *b*. *gambiense* group 1 strains that determine sensitivity to these primate lytic factors.

### Localisation of *P*. *papio* APOL1

**Fig 4:**
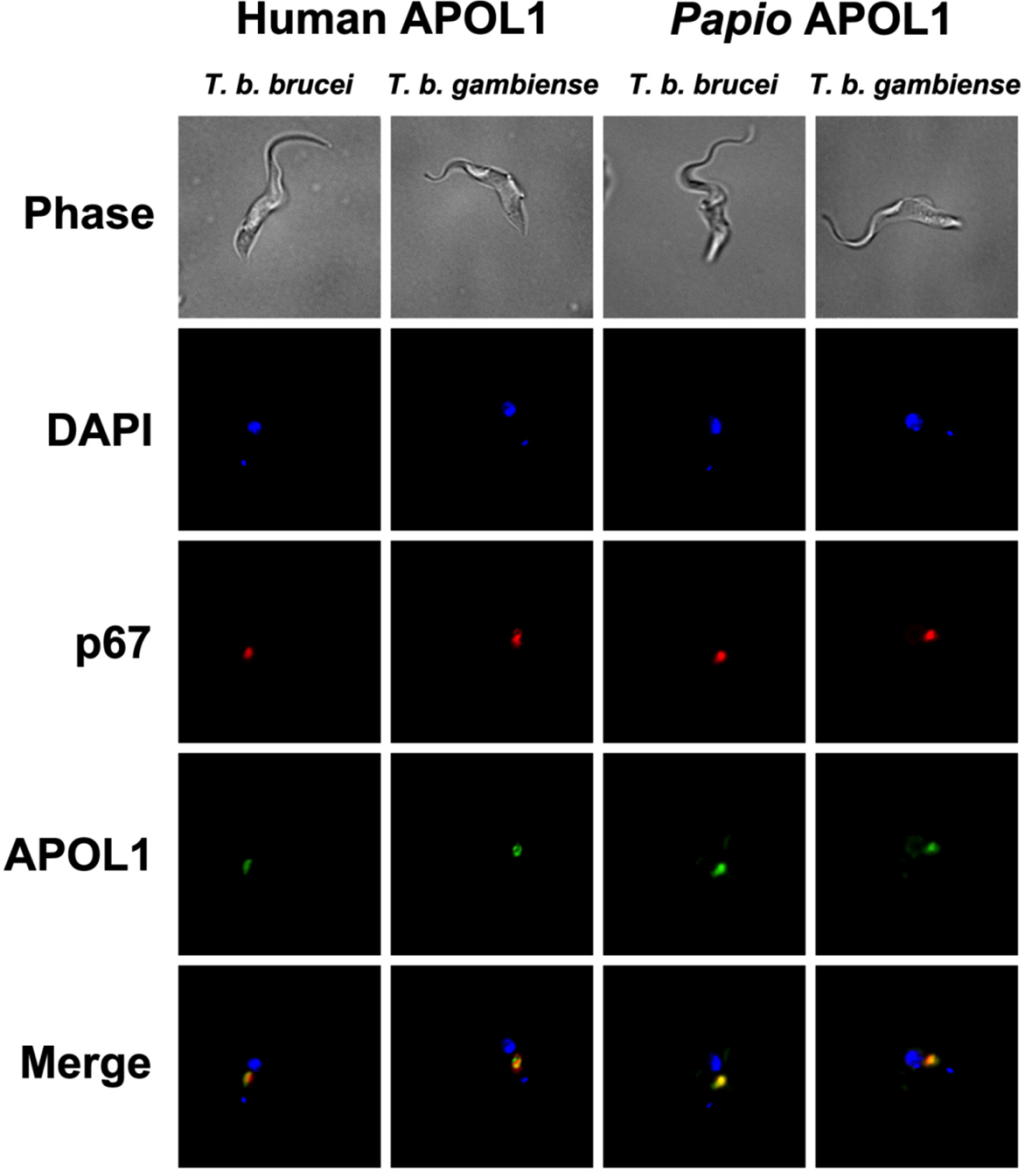
Recombinant APOL1 uptake and localisation. The localisation of Alexa488 (green) labelled anti-pentaHis antibody (APOL1), AlexaFluor594 (red) labelled anti-p67 (lysosomal membrane protein) and DAPI after a two-hour exposure to recombinant Human and *P*. *papio* APOL1 featuring an N-terminal 6xHis-tag. The panels represent human serum sensitive *T*. *b*. *brucei*, strain STIB247, and human serum resistant *T*. *b*. *gambiense* group 1, strain ELIANE.

Human recombinant APOL1 is taken up by fluid phase endocytosis and trafficked through the endocytic pathway to the endolysosome, the initial activation site of APOL1, in all *T*. *brucei* sub-species [27,48]. This results in lysis of *T*. *b*. *brucei* but not of *T*. *b*. *rhodesiense* or *T*. *b*. *gambiense* [48], which each possess mechanisms to resist the lytic effects of APOL1 [35,48,51,52]. To determine if *P*. *papio* APOL1 is localised through the parasite endolysosome pathway in a similar manner to that demonstrated for human APOL1, uptake of both recombinant proteins was compared in *T*. *b*. *brucei* and *T*. *b*. *gambiense* group 1 parasites using a fluorescent antibody to detect the His-tagged recombinant APOL1 protein. The cells were then examined by microscopy, in conjunction with the lysosomal marker p67. In order to achieve images of APOL1 uptake we used high concentrations of APOL1 (material and methods) to counteract possible degradation of APOL1 in the lysosome. Consistent with previous experiments of serum and APOL1 uptake in our laboratory [48,49,51], no lysosomal swelling was observed. As shown in Fig 4, both human and *P*. *papio* APOL1 are internalised by *T*. *b*. *brucei* and *T*. *b*. *gambiense* after a two hour incubation and are observed to co-localise with an antibody directed against the lysosomal membrane protein p67, indicative of the parasite endolysosome pathway [84,85]. These observations, in parallel with the ablation of lysis observed after co-incubation with acidotropic agent, ammonium chloride in APOL1 lysis assays, suggest that as previously demonstrated for human APOL1, exposure of the protein to the low pH of the endolysosomal pathway is also a requirement for trypanolytic activity of the baboon APOL1 ortholog.

## Discussion

The ancient co-evolutionary engagement of African trypanosomes with their mammalian hosts has shaped an innate lytic molecule in man that protects from infection with most African trypanosomes. In response, the extensive antigenic repertoire of *T*. *brucei* [86] has provided a rich resource from which to evolve counter-measures to APOL1-mediated lysis on at least two occasions; *SRA* in *T*. *b*. *rhodesiense* in East Africa [35,36,87], and *TgsGP* in *T*. *b*. *gambiense* group 1 in West Africa [51,52,88]. In this study we present a novel APOL1 variant from a species of West African baboon that killed examples of all *T*. *brucei* sub-species, including *T*. *b*. *rhodesiense*, *T*. *b*. *gambiense* group 2, and *T*. *b*. *gambiense* group 1, the agent of most current cases of human African trypanosomiasis. The identification of such genetic variants, capable of killing both animal and human-infective parasites presents new opportunities for unconventional approaches to disease treatment and control, using APOL1-based biological therapies.

Previous studies have identified *APOL1* orthologs in a subset of Old World monkeys [14,62], and an *APOL1* variant with a key similarity in some humans with African ancestry [63], that encode proteins lytic to *T*. *b*. *rhodesiense*. In both variants, evidence suggests protection is mediated by the position of a single lysine residue in the C-terminal protein domain that obstructs coiled-coil interactions with SRA, thus allowing APOL1-directed lysis to proceed unimpeded [62]. Unfortunately in humans, the two amino acid deletion that alters the SRA-binding region in this APOL1-G2 variant come with an associated fitness cost: a 7–29-fold increased risk of developing a wide spectrum of kidney disorders in individuals carrying two copies of a variant allele [63,89–92]. The exact biological mechanism underlying this APOL1-associated nephropathy is not yet known but appears to be specific to the human variants. Engineered versions of the human APOL1 variant transiently expressed in a mouse model caused significant toxicity to the organ of expression (liver), which was not observed with baboon APOL1 or human APOL1 modified to introduce only the protective baboon lysine to the C-terminus [62]. This is an encouraging result, and such baboon-like APOL1 variants are now the focus of efforts to create suitable mechanisms of delivery, such as the conjugation of APOL1 protein to an antibody fragment targeted to parasite surface antigens [93] and an ambitious project to create targeted transgenic cattle expressing variant APOL1 [15].

These variants could be used to protect the reservoir host species from zoonotic *T*. *b*. *rhodesiense* sleeping sickness in addition to animal trypanosomiasis, which places severe restrictions on agricultural production and rural development in Sub-Saharan Africa [1]. Unfortunately, they will have a limited effect on the overall burden of human sleeping sickness. None of the APOL1 variants used in these experiments are able to kill the major human pathogen *T*. *b*. *gambiense* group 1 which places a population of 57 million people in West and Central Africa at risk of disease [4], less than 5% of whom are currently under surveillance [94]. Furthermore, there is a risk that the proposed interventions could result in the creation of a vacant ecological niche that increases the incidence of *T*. *b*. *gambiense* group 1 in domestic livestock through selective removal of susceptible competitor species such as *T*. *b*. *brucei*, *T*. *congolense*, *T*. *vivax* and *T*. *b*. *rhodesiense*.

We have addressed these concerns directly in this study by examining the serum of a West African baboon species *P*. *papio* that overlaps in distribution with that of *T*. *b*. *gambiense*, and which had been suggested to self-cure *T*. *b*. *gambiense* group 1 infection [64]. In that study primates infected with *T*. *b*. *gambiense* group 1 parasites exhibited a serological response that decreased throughout the course of the experiment and had no detectable parasitemia, consistent with an initial infection, followed by rapid parasite clearance and self-cure. In our study *P*. *papio* serum is able to lyse *T*. *b*. *gambiense* in 24 hours *in vitro*. The difference in timing of parasite killing between the *in vivo* and *in vitro* experiments, which could be due to a number of different factors such as parasite sequestration, is a well-recognised phenomenon. It is possible that parasites avoid lysis by residing in sites of low APOL1 concentrations (for example at the bite site in the skin) in the animal before eventually being cleared. This factor must be taken into account when attempting to develop APOL1-based therapies as *in vitro* assays do not always reflect the complexity of *in vivo* cell biology. The introduction of improved bioluminescent imaging to quantify parasite burden could be used to test *in vivo* for complete parasite clearance.

We have shown that the lytic effect of *P*. *papio* serum can be reproduced with an ortholog of the trypanolytic primate defence protein, APOL1, which demonstrates the uptake and localisation characteristics of other previously identified APOL1 proteins [27,48]. The trypanolytic action of this *P*. *papio* APOL1 variant against *T*. *b*. *rhodesiense* can be attributed to the C-terminal lysine mutation that is conserved among several members of the Cercopithecine subfamily that includes baboon, mandrills and mangabeys [62]. However the mechanism by which it counters *T*. *b*. *gambiense*, which has evolved multiple contributing mechanisms of human serum resistance, remains more elusive. All *T*. *b*. *gambiense* group 1 parasites share a mutated *HpHbR* with reduced affinity for one of the human APOL1-containing particles (TLF-1) via the HPR ligand [48–50], although a second particle, TLF2, appears to have alternative, as yet unresolved, mechanism(s) of internalisation [24,80,81,83]. The exact composition of TLF in baboon serum has not been clarified. However analysis of an HPR-affinity purified HDL sub-fraction from *P*. *hamadryas* baboon serum detected a TLF-equivalent particle that contains the same structural components as human TLF [14]. Furthermore, when transiently expressed in mice, all three components were required for maximum lytic activity against *T*. *b*. *rhodesiense* [14], suggesting HPR-HpHbR may play a role in uptake of baboon TLF. Here we show that *T*. *b*. *gambiense* group 1, although still fully susceptible to sub-physiological concentrations of *P*. *papio* serum, was 1000-fold less sensitive than *T*. *b*. *brucei* sub-species. This difference was ablated when functional *T*. *b*. *brucei* HpHbR was restored to the *T*. *b*. *gambiense* parasite, supporting a role for *P*. *papio* TLF uptake via both HpHbR-mediated endocytosis as well as unidentified alternative mechanisms, possible shared with those already proposed for human TLF [24,80,81,83].

Secondly, the *TgsGP* gene has been demonstrated to be essential for human serum resistance in *T*. *b*. *gambiense* group 1, as gene deletion renders the parasites sensitive to human serum lysis [51,52]. In contrast to the *T*. *b*. *rhodesiense* SRA protein, TgsGP and APOL1 do not appear to interact directly. Instead, TgsGP is proposed to bolster *T*. *b*. *gambiense* resistance to human APOL1 pore-forming activity through a process of plasma membrane stiffening [52]. A third mechanism by which *T*. *b*. *gambiense* might resist the actions of NHS, through enhanced APOL1 degradation within the endolysosomal system, has also been proposed [52]. Modulation of expression levels of the cysteine protease Cathepsin L and its inhibitor (ICP) has demonstrated an important role for cathepsin-mediated degradation of APOL1 in human serum resistance [53]. Difference in expression levels of these genes has not been detected in *T*. *b*. *gambiense*, however a lower pH is observed within the early endosomes that is predicted to accelerate their proteolytic activity relative to *T*. *b*. *brucei* [52]. Intriguingly, we observed equal sensitivity of all strains tested to *P*. *papio* APOL1-directed lysis, suggesting that the activity of TgsGP, and APOL1 degradation by cysteine peptidases, that effectively hinders human APOL1 in *T*. *b*. *gambiense*, poses no such barrier to the *P*. *papio* variant. This raises interesting questions about how exactly *P*. *papio* APOL1 is able to overcome these factors? Many details of the action of the TgsGP protein in particular remain cryptic. Despite its essential role in human serum resistance in *T*. *b*. *gambiense*, ectopic expression of *T*. *b*. *gambiense* TgsGP alone in *T*. *b*. *brucei* is insufficient to confer resistance to human serum [51,88]. There is evidently a role for other, as yet unidentified processes, in *T*. *b*. *gambiense* human serum resistance, which are absent or incomplete in *T*. *b*. *brucei*.

Sequence analysis has revealed that baboon and human APOL1 orthologs share only 58% amino acid sequence identity [14]. Despite this, in the recently elucidated example of baboon serum lysis of *T*. *b*. *rhodesiense* it was demonstrated that a single amino acid substitution conserved between baboon species is responsible for APOL1 evasion of SRA binding [62]. Uncovering the mechanism by which *P*. *papio* has developed its broad trypanolytic ability may offer further insights into the workings of *T*. *b*. *gambiense* human serum resistance, as well as aid in the design of an improved APOL1 therapy that could target all pathogenic trypanosomes across Sub-Saharan Africa. Such universal therapies that can treat both animal and human pathogens are particularly appropriate to the “one health” approach, currently advocated by WHO, FAO, and OIE, that integrates medical and veterinary health policy and research for addressing zoonotic diseases.

The Guinea baboon *P*. *papio* is found only in a limited area of western equatorial Africa, where its range overlaps with that of *T*. *b*. *gambiense* group 1. Five other baboons are represented in the *Papio* genus of which serum for only one, the east African *P*. *cynocephalu*s (yellow baboon) has been previously tested against *T*. *b*. *gambiense* parasites, and was reported to be non–lytic [37]. Unfortunately APOL1 sequence is currently unavailable for comparative analysis with this species or the southern African *P*. *ursinus* (Chacma baboon) and *P*. *kindae* (Kinda baboon) species from Central Africa. Of the remaining *Papio* species, APOL1 sequences from cDNA have been successfully obtained for *P*. *hamadryas* (Hamadryas baboon) from North East Africa, and Central African *P*. *anubis* (Olive baboon) [62], the closest related species to *P*. *papio* in a recent phylogenetic study of mitochondrial DNA [95]. Amino acid alignments of *P*. *papio* APOL1 with these available sequences indicate ~98.5% identity to *P*. *hamadryas* and 93.5% to *P*. *anubis* (S3 Fig). A study in which C-terminal polymorphisms of *P*. *anubis* were incorporated into human recombinant APOL1 were observed to be lytic to *T*. *b*. *rhodesiense* but not *T*. *b*. *gambiense* [37], however full length APOL1 transcripts, unavailable at the time of the study, have not been tested. For *P*. *hamadryas*, serum and APOL1 have not yet been tested against *T*. *b*. *gambiense*, however a laboratory infection of two individual baboons with a strain of *T*. *b*. *gambiense* group 1 suggested hamadryas baboons to display a level of trypanotolerance to infection [64]. Future studies in which the sensitivity of *T*. *brucei* subspecies to serum and APOL1 from the other baboon species, followed by the construction of chimera mutants are now needed to help resolve the crucial polymorphisms responsible for *T*. *b*. *gambiense* lysis, as has been successful for *T*. *b*. *rhodesiense*.

## Supporting Information

S1 Fig Purification of recombinant human and *P*. *papio* APOL1 protein variants. APOL1 variants were produced in *E*. *coli*, based on *APOL1* cDNA sequence *H*. *sapiens* (accession no. CCDS13926.1) and *P*. *papio* (accession no. KC197810), minus the N-terminal signal peptide (*H*. *sapiens*, residues 28–398; *P*. *papio*, residues 28–288) and with the addition of an N-terminal 6xHis-tag. Proteins were purified using Ni-Sepharose under denaturing conditions and dialyzed against 20mM acetic acid and 0.05% tween. Purity and concentration of the final purified protein was checked using a Qubit fluorometer (Thermofisher) and SDS-PAGE (P = *P*. *papio*, H = human APOL1) stained with Brilliant blue G solution (Sigma-Aldrich) alongside SeeBlue Plus2 protein standards (Thermofisher). APOL1 concentration was adjusted to 1 mg/ml and stored in aliquots at 4°C.
(TIF)

S2 Fig Trypanolytic activity of serum from an adult male Guinea baboon (*P*. *papio)* against representative examples of *T*. *brucei* sub-species. To confirm the lytic ability of *P*. *papio* sera, the percentage of viable trypanosomes was determined following a 24-hour exposure to serial dilutions of an alternative Guinea baboon serum, sourced from an individual adult male (Matrix Biologicals, UK). Representative *T*. *brucei* sub-species strains were tested: *T*. *b*. *brucei* (strain STIB247), *T*. *b*. *rhodesiense* (strain EATRO98), *T*. *b*. *gambiense* group 1 (strain ELIANE) and *T*. *b*. *gambiense* group 1 expressing a functional *T*. *b*. *brucei* HpHbR receptor (ELIANE *TbbHpHbR* ^*-/+*^). Mean percentage cell survival ± SD is expressed relative to FBS control, calculated from three independent experiments. Dose–response curves and IC_50_ values with 95% confidence intervals (CI) were determined using GraphPad Prism software version 7.
(TIF)

S3 Fig Amino acid alignment of Human and baboon APOL1 orthologs. APOL1 amino acid sequence of the Old World monkey baboon species, *Papio papio*, was aligned with *P*. *anubis*, *P*. *hamadryas* and human (*Homo sapiens*) sequences. Dashes represent gaps introduced into the alignment by nucleotide deletions, and shading indicates amino acid differences. The position of the lysine (K) residue in the APOL1 C-terminus, implicated in resistance to *T*. *b*. *rhodesiense*, and present in several species of Old World monkey and the G2 APOL1 human variant is indicated (arrowhead).
(TIF)

S4 Fig Trypanolytic activity of recombinant *P*. *papio* APOL1 protein against additional strains of *T*. *b*. *gambiense* group 1. The percentage of viable trypanosomes was determined following a 24-hour exposure to media containing serial dilutions of ***P*. *papio*** recombinant APOL1 protein. ***T*. *b*. *gambiense*** group 1 strains (ELIANE, TOBO and S1/1/6 [Côte d'Ivoire], PA [Republic of the Congo], and BIM [Cameroon]) were tested. The mean percentage cell survival ± SD, relative to protein-free control, was calculated from at least three independent experiments. Dose–response curves were determined using GraphPad Prism software version 7.
(TIF)
