# Supplementary figures and images for: A Primate APOL1 Variant That Kills Trypanosoma brucei gambiense
Source: PLoS Negl Trop Dis. 2016 Aug 5;10(8):e0004903. doi: 10.1371/journal.pntd.0004903 (PMC4975595; doi:10.1371/journal.pntd.0004903)

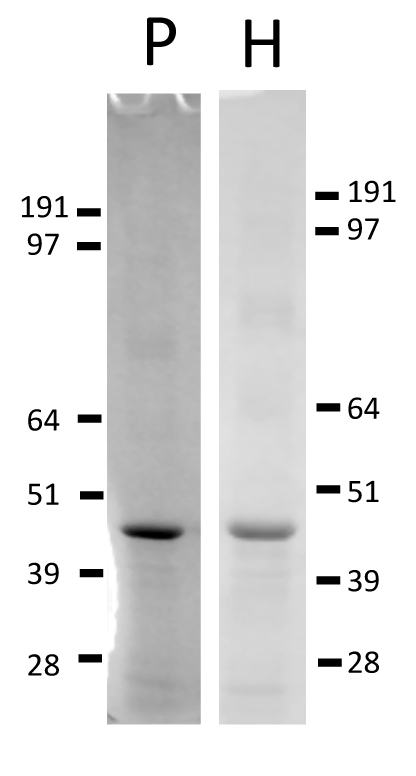

Supplement: S1 Fig — APOL1 variants were produced in E. coli, based on APOL1 cDNA sequence H. sapiens (accession no. CCDS13926.1) and P. papio (accession no. KC197810), minus the N-terminal signal peptide (H. sapiens, residues 28–398; P. papio, residues 28–288) and with the addition of an N-terminal 6xHis-tag. Proteins were purified using Ni-Sepharose under denaturing conditions and dialyzed against 20mM acetic acid and 0.05% tween. Purity and concentration of the final purified protein was checked using a Qubit fluorometer (Thermofisher) and SDS-PAGE (P = P. papio, H = human APOL1) stained with Brilliant blue G solution (Sigma-Aldrich) alongside SeeBlue Plus2 protein standards (Thermofisher). APOL1 concentration was adjusted to 1 mg/ml and stored in aliquots at 4°C. (TIF) [file pntd.0004903.s001.tif]

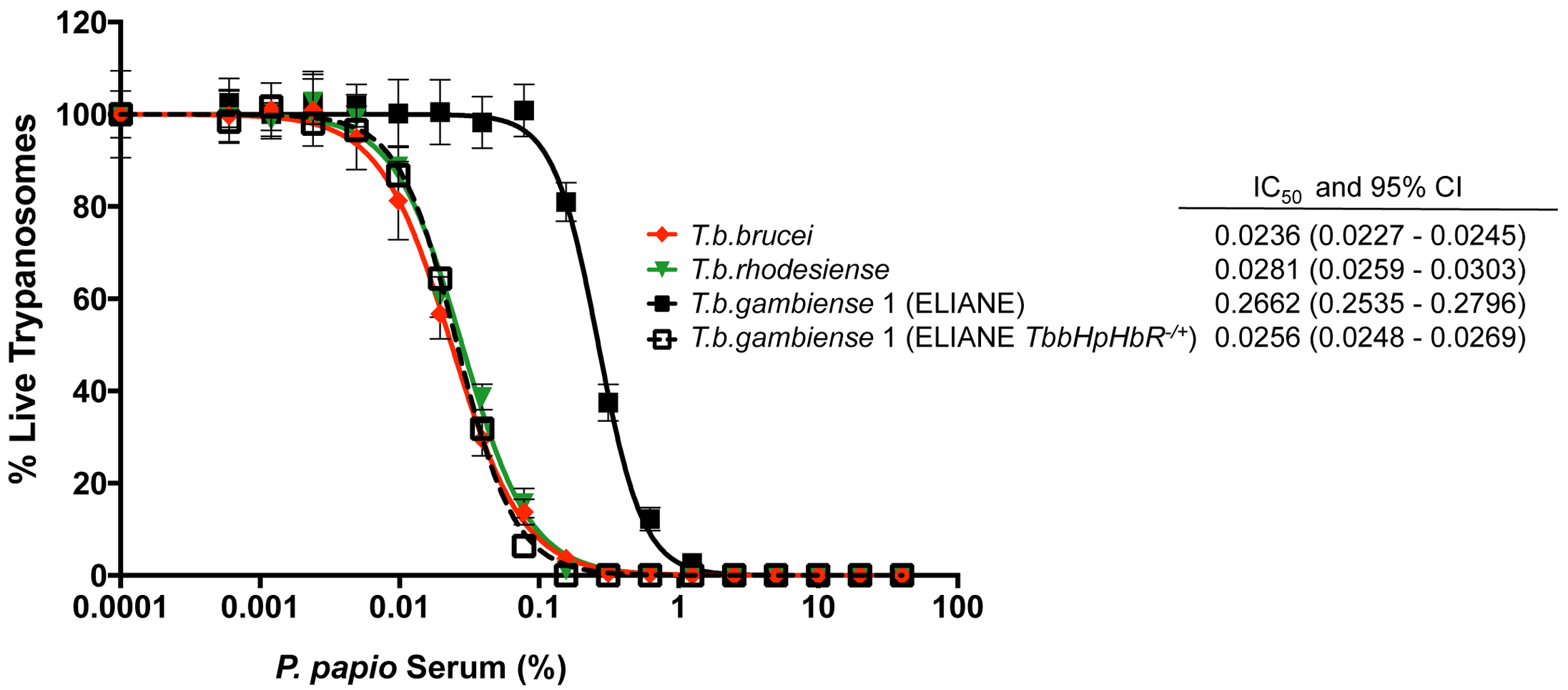

Supplement: S2 Fig — To confirm the lytic ability of P. papio sera, the percentage of viable trypanosomes was determined following a 24-hour exposure to serial dilutions of an alternative Guinea baboon serum, sourced from an individual adult male (Matrix Biologicals, UK). Representative T. brucei sub-species strains were tested: T. b. brucei (strain STIB247), T. b. rhodesiense (strain EATRO98), T. b. gambiense group 1 (strain ELIANE) and T. b. gambiense group 1 expressing a functional T. b. brucei HpHbR receptor (ELIANE TbbHpHbR -/+). Mean percentage cell survival ± SD is expressed relative to FBS control, calculated from three independent experiments. Dose–response curves and IC50 values with 95% confidence intervals (CI) were determined using GraphPad Prism software version 7. (TIF) [file pntd.0004903.s002.tif]

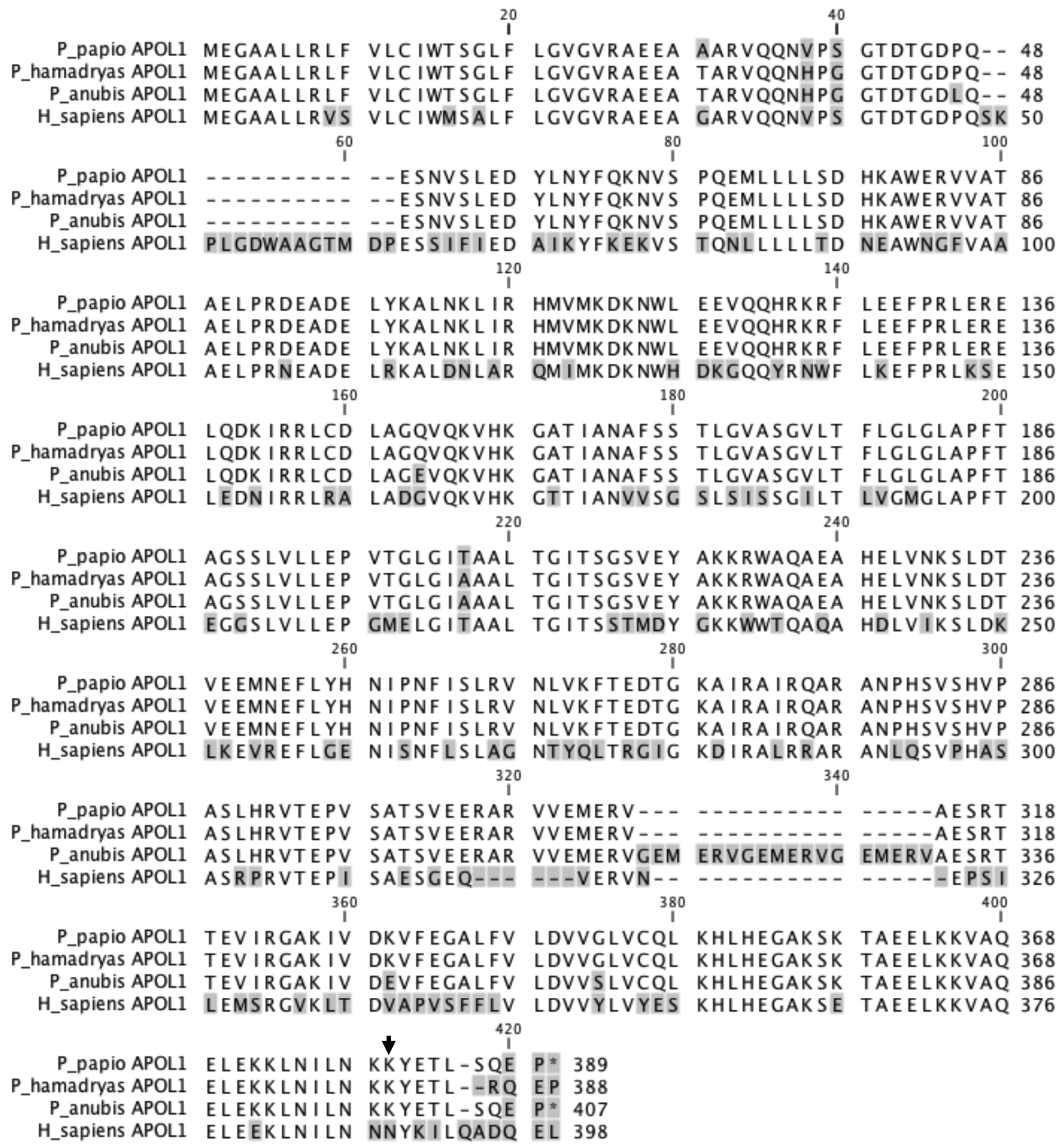

Supplement: S3 Fig — APOL1 amino acid sequence of the Old World monkey baboon species, Papio papio, was aligned with P. anubis, P. hamadryas and human (Homo sapiens) sequences. Dashes represent gaps introduced into the alignment by nucleotide deletions, and shading indicates amino acid differences. The position of the lysine (K) residue in the APOL1 C-terminus, implicated in resistance to T. b. rhodesiense, and present in several species of Old World monkey and the G2 APOL1 human variant is indicated (arrowhead). (TIF) [file pntd.0004903.s003.tif]

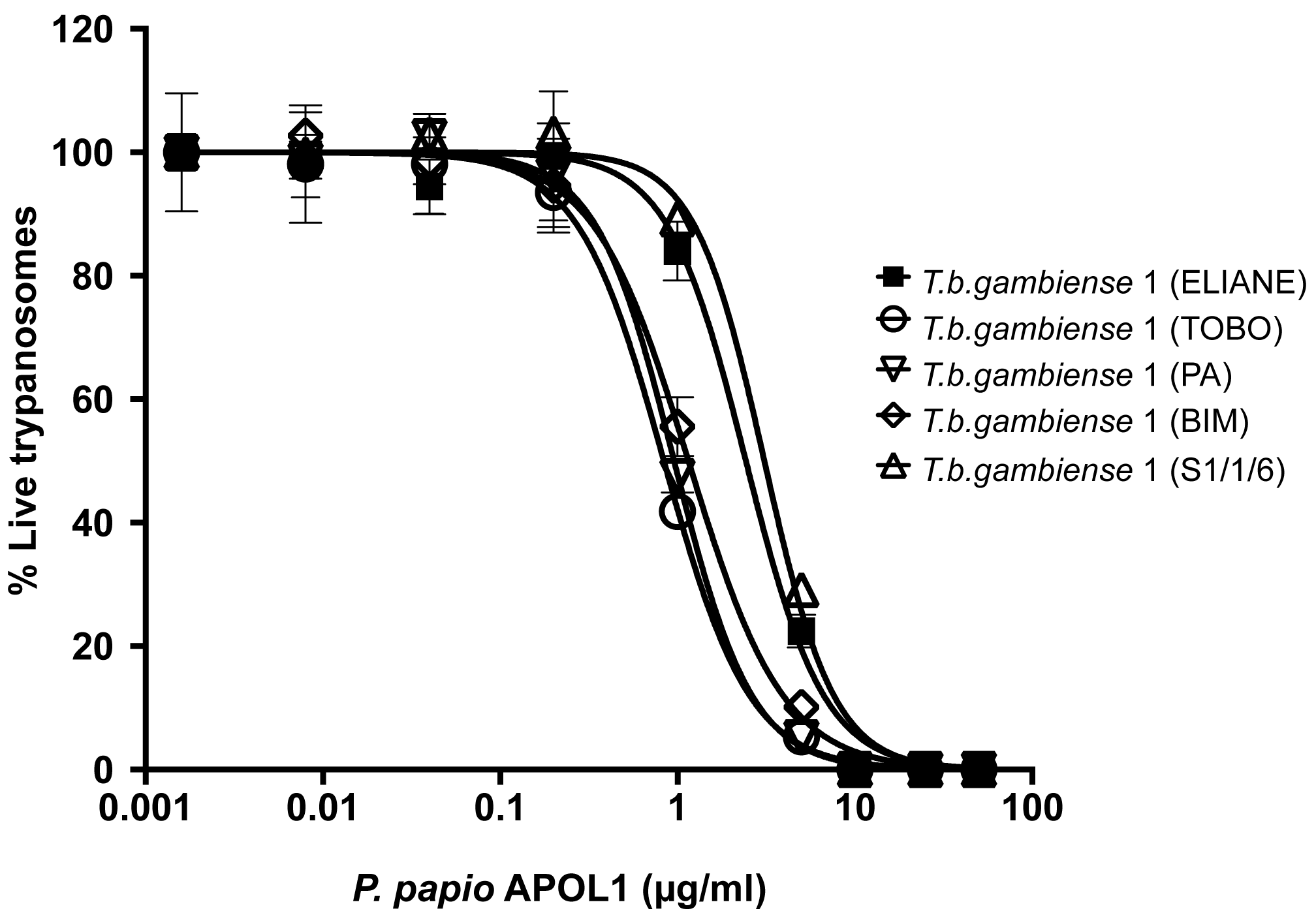

Supplement: S4 Fig — The percentage of viable trypanosomes was determined following a 24-hour exposure to media containing serial dilutions of P. papio recombinant APOL1 protein. T. b. gambiense group 1 strains (ELIANE, TOBO and S1/1/6 [Côte d'Ivoire], PA [Republic of the Congo], and BIM [Cameroon]) were tested. The mean percentage cell survival ± SD, relative to protein-free control, was calculated from at least three independent experiments. Dose–response curves were determined using GraphPad Prism software version 7. (TIF) [file pntd.0004903.s004.tif]
